# Detection and investigation of atypical porcine pestivirus in a swine production system

**DOI:** 10.3389/fvets.2022.998344

**Published:** 2022-10-11

**Authors:** Grace E. Houston, Cassandra K. Jones, Jason C. Woodworth, Rachel Palinski, Chad B. Paulk, Tom Petznick, Jordan T. Gebhardt

**Affiliations:** ^1^Department of Diagnostic Medicine/Pathobiology, College of Veterinary Medicine, Kansas State University, Manhattan, KS, United States; ^2^Department of Animal Sciences and Industry, College of Agriculture, Kansas State University, Manhattan, KS, United States; ^3^Department of Grain Science and Industry, College of Agriculture, Kansas State University, Manhattan, KS, United States; ^4^ArkCare, Norfolk, NE, United States

**Keywords:** atypical porcine pestivirus (APPV), investigation, persistence, production system, swine

## Abstract

A commercial farrow-to-finish farm was suspicious of atypical porcine pestivirus (APPV) after observing clinical signs of congenital tremors (CT) and splay leg (SL) of newborn pigs. If introduced onto the farrow-to-finish, the two potential routes of introduction could be through replacement gilts or incoming semen doses. Therefore, this study aimed to determine the prevalence of clinical APPV within the sampled population, identify the route of APPV introduction to this system, and determine prevalence of detectable APPV RNA within a population of gilt multiplication farm offspring through an isolation nursery and finisher barn. Farrowing records were analyzed for the presence of CT or SL and corresponding parity of the dam. Overall, prevalence of clinically affected litters within batch farrowing groups ranged from 0 to 31%. Phylogenetic analysis was conducted on a serum sample from a gilt at the isolation nursery, semen dose for the farrow-to-finish farm, and serum of a CT piglet. Results indicated that the virus circulating in clinically affected piglets was most similar to an incoming semen dose (98.9% nucleotide identity). Blood samples were collected at four time points and revealed APPV clinical prevalence was 37.5–77.5% during the nursery phase and 0–26% during the finisher phase. Oral fluids were also collected during the finisher phase and APPV clinical prevalence was 100% for all sampling time points. In summary, introduction of APPV into naïve herds is associated with increased clinical CT and SL cases and is detectable in asymptomatic pigs during the nursery and finisher production phases. This study found that potential screening tests for APPV could include oral fluids or qRT-PCR analysis of semen doses especially when trying to identify prevalence levels on naïve farm.

## Introduction

Atypical porcine pestivirus (APPV) was first identified in the United States in 2015. It is a Flavivirus linked to congenital tremors (CT) and splay-leg (SL) in pigs (1–3). The CT is characterized by muscle spasms in the head and body that can persist for weeks to months, typically diminishing by marketing (4, 5). While SL is a temporary dysfunction of the posterior legs following birth (2–4). Preweaning mortality associated with CT and SL is most often associated with inadequate feeding or difficulty standing or moving rather than the condition itself (5).

Atypical porcine pestivirus targets the cerebellum and lymph nodes, but has also been detected in feces, boar preputial swabs, preputial fluid, and semen (6–8). In 2018, APPV molecular prevalence was 28.7% in the Midwestern United States (US) and notably higher in states with the greatest swine production - IA, 30.6%; IL, 32.9%; MN, 37.4% (9). Atypical porcine pestivirus is associated with transient or persistent infections in asymptomatic pigs promoting global dissemination (10). Furthermore, APPV is highly mutable producing many genetically divergent strains (9). For this report, a commercial farrow to finish production system observed an increase in CT and SL. Serum was collected from clinically affected piglets, submitted to the Kansas State Veterinary Diagnostic Laboratory (KSVDL), and was found to have detectable APPV RNA via qRT-PCR analysis. The objectives of this study were to determine the prevalence of clinical APPV on the farrow-to-finish farm, determine the route of introduction onto the farm, and determine if APPV persisted within asymptomatic pigs co-housed with gilts intended for the farrow-to-finish farm and their environment.

## Materials and methods

All blood sampling and oral fluid collection were approved by Kansas State University institutional animal care and use committee (IACUC) protocol #4457.

### Case history

A commercial farrow-to-finish farm located in Central Kansas was used in this experiment. The farm batch farrows 30 sows every 35 days. Gilts and sows are stalled until confirmed pregnant, then moved to group housing. From December 2019 to June 2020, 1–2 litters per batch exhibited CT or SL. The prevalence rates of CT and SL increased in July 2020, which led to suspicion of APPV. Serum samples from CT piglets and two semen samples were submitted to KSVDL for APPV qRT-PCR testing. The APPV qRT-PCR assay utilized and made commercially available is based on the assay described by Yuan et al. (11). For this assay, a Ct < 37 is considered positive for APPV RNA, suspect between 37 and 39, and negative for APPV RNA with a Ct above 39. Serum samples from CT piglets had a cycle threshold (Ct) value of 26.3–28.5 while one of the two semen samples had a Ct value of 30.5. Atypical porcine pestivirus was a likely diagnosis given the clinical picture and APPV qRT-PCR results. However, to confirm a diagnosis of APPV given the absence of confirmation through histopathology of tissue, one of the CT piglet serum samples was submitted for metagenomics. Metagenomics analysis indicated a low number of influenza and porcine reproductive and respiratory syndrome virus reads, significant porcine endogenous retrovirus reads, and a diversity of bacteria, including Proteobacteria, Clostridium, Bacillus, and Mycoplasma. As none of the identified organisms fully explained the clinical signs, APPV was deemed the likely cause of CT and SL in newborn pigs at this facility.

**Figure 1:**
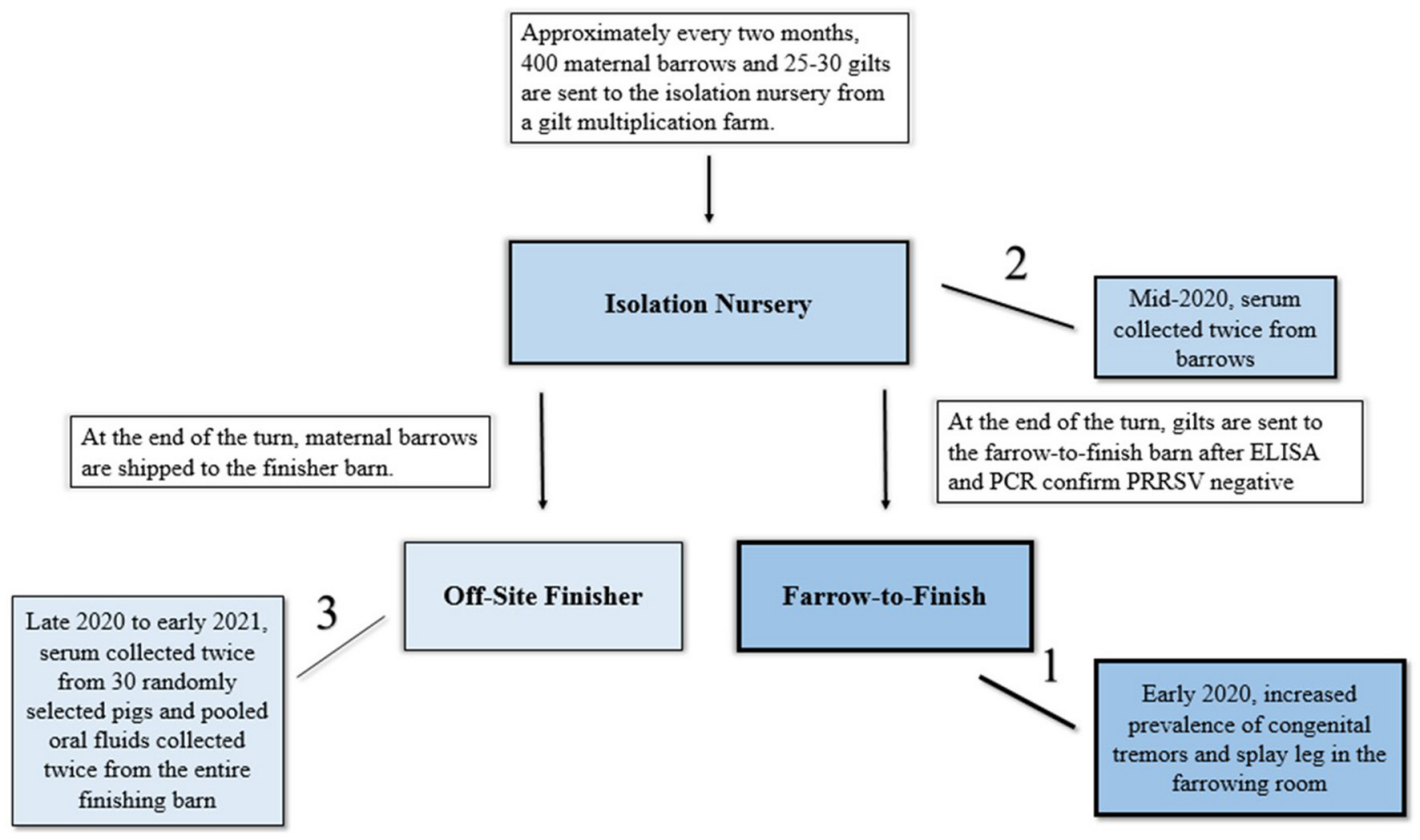
Depiction of the relationship between the isolation nursery barn, the farrow-to-finish site, and off-site finisher barn and timeline of events during this investigation of atypical porcine pestivirus persistence (APPV) in pigs. Arrows indicate movement of pigs while lines indicate order of events of sampling which corresponds to the shading of blue in the graphic.

The commercial farrow-to-finish farm receives gilts from an isolation nursery located off site. This isolation nursery receives weaned gilts and barrows from a gilt multiplication facility every 2 months (Figure 1). The barrows in the isolation nursery, at the end of the production turn, are shipped to a separate finisher facility while the gilts are transported to the commercial farrow-to-finish farm. Prior to transport, all gilts are tested for porcine reproductive and respiratory syndrome virus RNA and antibodies and negative gilts are introduced onto the commercial farrow-to-finish farm. In August 2020, following the CT/SL outbreak on the commercial farrow-to-finish farm, 5 randomly selected barrows were bled and serum submitted for APPV qRT-PCR 24-h after placement into the isolation nursery. Two of the five barrows had detectable APPV RNA (Ct values of 27.5 and 30.4). Given that there was detectable APPV RNA in the isolation nursery and the connection to the commercial farrow-to-finish facility, the isolation nursery was deemed as a potential source of APPV introduction. During the entirety of the study, no clinical signs of CT or SL were noted at the isolation nursery.

### Farrowing data record analysis

Farrowing records from January 10, 2019 to March 2, 2021 were analyzed for the prevalence of CT or SL litters. If a comment of “shakers” or “splays” was on the farrowing card, the litter was included in litter prevalence and divided by the total number of females that farrowed during the specified batch. Parity information was also analyzed from the same farrowing cards. If the farrowing card indicated the parity of the female as “1,” these females were designated as gilts, while all other parities were designated as sows.

### Environmental sampling

Environmental sampling using 10 × 10 cm cotton gauze was conducted similar to Elijah et al. (12). Briefly, environmental swabs were taken after the isolation nursery was sanitized but before new pigs were placed at the location. A total of 27 sample sites were identified at the farm, including entryways into barns, changing areas for employees, boots utilized by workers, and floors of selected pens. It was chosen to focus on areas of direct pig contact and potential fomites given evidence to suggest that fomites play an important role in the transmission of this disease (4). If a site was selected for environmental swabbing, a clean pair of gloves was donned and a 10 × 10 cm cotton surgical gauze square pre-moistened with 5 mL of phosphate-buffered solution (PBS) was used to sample the area. Environmental samples were stored in an −80°C freezer prior to submission. For submission, five of 27 environmental samples were chosen at random, defrosted, 20 mL of PBS added to the environmental sample, manually agitated for 5–10 s, and analyzed for APPV RNA. For submission, all five selected environmental samples were from pen floors that had direct pig contact.

### Blood and oral fluids collection

After the initial confirmation of APPV, serum from the barrow population was collected twice in isolation nursery and twice in the finisher barn. For the first isolation nursery sampling date, 200 barrows were individually bled. Forty barrows were removed from the facility as part of a concurrent research trial after this point, so 160 barrows were individually bled in the second sampling date. For each collection date in the isolation nursery, serum samples were pooled and submitted for APPV testing (5 pigs/pen/pool). When a positive APPV qRT-PCR was identified in the pooled samples, a subset of individual pig serum were submitted. In the finishing barn, there were 2 large pens that can accommodate 150 animals per pen. Upon arrival to the finishing barn, all barrows from the isolation nursery are allocated between the two large pens. For serum sampling during the finisher phase, 15 pigs were randomly selected from each pen. For oral fluid collection, five ropes were hung in each pen and evenly dispersed throughout the pen so that all pigs within the pen could chew on oral fluid ropes. Oral fluid and serum samples collected from the finisher barn were submitted for APPV qRT-PCR.

### Sequencing and phylogenetic analysis

Atypical porcine pesitvirus qRT-PCR positive samples were submitted for APPV E2 sequencing at the KSVDL. Submitted samples included one piglet serum sample from the farrow-to-finish farm (Ct = 26.3), one semen dose intended for artificial insemination of farrow-to-finish gilts or sows (Ct = 30.5), and one replacement gilt serum sample from the isolation nursery (Ct = 27.5). All three sequences were submitted to Genbank prior to publication; piglet sample, semen dose, and gilt sample can be accessed by their respective Genbank accession number as follows: ON651441, ON651443, and ON651442.

Viral RNA was extracted with the MagMax viral RNA Isolation kit (Thermo Fisher) on a Kingfisher platform. Amplicons were generated from viral RNA using Superscript III One-Step RT-PCR System with Platinum Taq and specific primers, using a 56°C annealing temperature and 30 s extension time. Amplicons were purified using the HighPrep PCR clean-up System, library prepped by Nextera XT v2 DNA Library prep kit and sequenced on an Illumina Iseq (300-cycle cartridge), as specified by the manufacturer. Raw reads were trimmed for quality and mapped to the closest reference (Genbank #MK728876). Consensus sequences were extracted from the mapping and used for subsequent analysis. All bioinformatics was performed in CLC workbench v20 using default parameters. The resulting testing generated a partial E2 sequence form the semen dose but complete E2 sequences from the replacement gilt and piglet samples.

Consensus sequence alignment was performed using MUSCLE (13) in MEGA-X v10.0. The evolutionary history was inferred by using the maximum likelihood method with the Tamura-Nei model (14) and evaluated with 1,000 bootstrap replicates (15). Cut off value for the consensus tree was set to 70%. Pairwise differences were computed in Mega X using the pairwise differences function (16).

## Results

**Figure 2:**
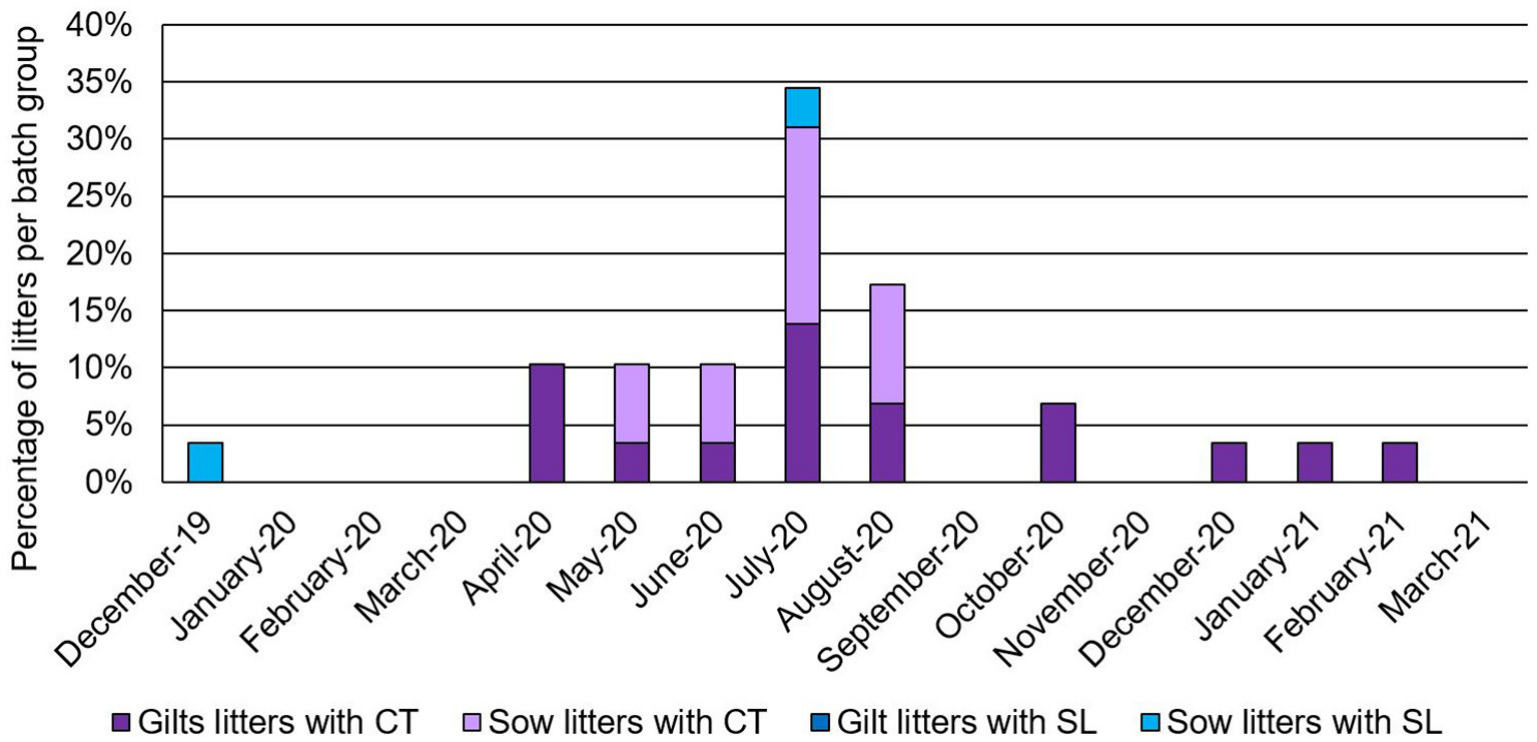
Percentage of litters from a farrowing batch group with clinical signs of congenital tremors (CT) or splay leg (SL) then break down of the litters with clinical cases based on parity (females considered a gilt if this was their first litter farrowed, all other females considered sows). Date is designated as month-year.

**Figure 3:**
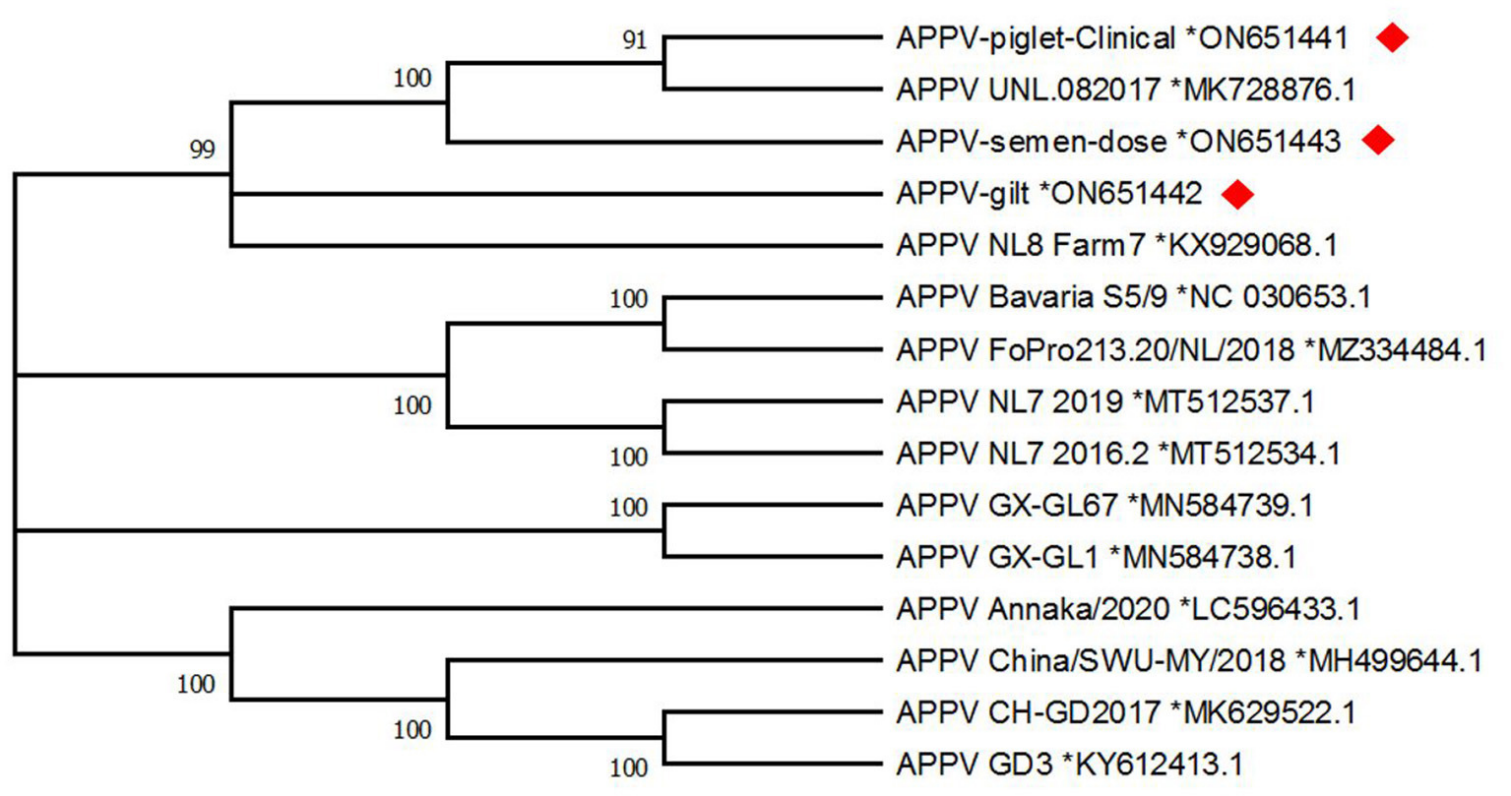
Maximum likelihood phylogenetic tree generated with 1,000 bootstrap replicates (MEGA-X) for atypical porcine pestivirus E2; evolutionary history was inferred by using the maximum likelihood method and Tamura-Nei model. Sequences from this study, designated with a red diamond were obtained from a semen dose, serum from a clinically affected piglet, and serum from an incoming gilt. Reference sequences are named by isolate name and Genbank reference number listed after the black asterisk. Frequencies for a branch that are below 70% are not displayed.

Prevalence of clinical APPV cases was indistinguishable between sows (prevalence of affected litters within batch farrowing group ranging from 4 to 17%; average of 2.96% of litters affected over sampling period; CT CI = 1.03–4.88%; SL CI = 0.04–0.94%) and gilts (prevalence of affected litters within batch farrowing group ranging from 4 to 14%; average of 3.94% of litters affected over sampling period; CT CI = 2.42–5.46%; Figure 2). In this study, APPV RNA was detected at both introduction points for the commercial farrow-to-finish farm. The boar semen and replacement gilt serum contained APPV RNA, however, the clinical piglet APPV E2 sequence was most similar to that of the boar semen (98.9 vs. 95.9% at the nucleotide level; Table 1). In addition, phylogenetic analyses clustered the gilt sequence in a separate clade than the semen and piglet sequences, further supporting the hypothesis that the boar semen and clinical piglet were more similar when compared to the gilt (Figure 3).

**Table 1:** Amino acid similarities (%) among atypical porcine pestivirus (APPV) E2 regions of 3 newly generated strains in this study.

**Strain Name**	**APPV-semen-dose**	**APPV-piglet-clinical**	**APPV-Gilt-sample**
APPV-semen-dose	–	–	–
APPV-piglet-clinical	98.77	–	–
APPV-Gilt-Sample	94.73	95.55	–

**Figure 4:**
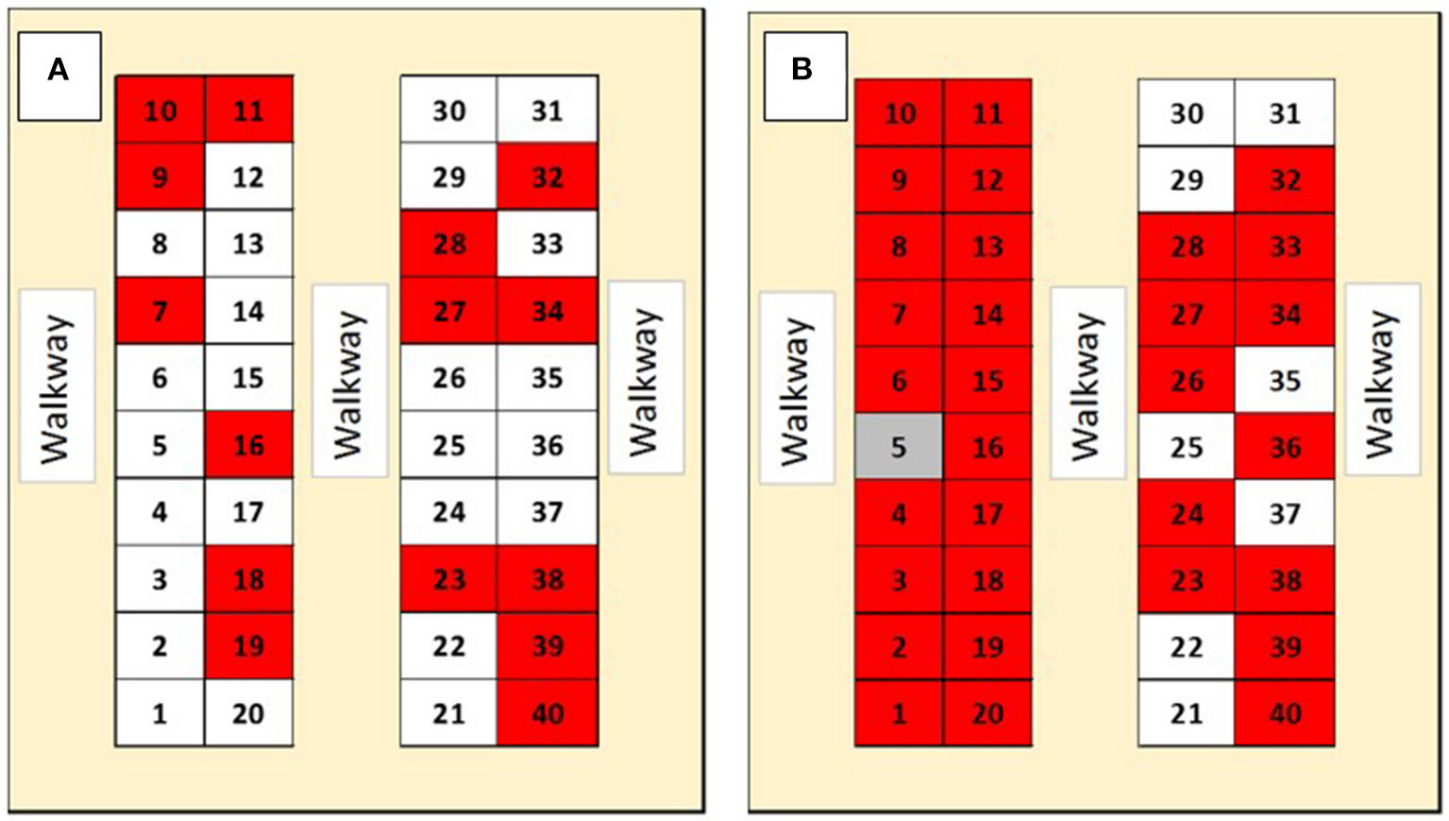
Prevalence of Atypical porcine pestivirus (APPV) RNAemia in serum samples pooled by pen in an isolation nursery facility. Numbers within figure illustrate pen location within each of two identical nursery facilities located on the same premise. The diagram on the left **(A)** is from sampling on 8/20/2020, while the diagram on the right **(B)** is from sampling on 9/30/2020. Gray indicates a suspected detectable APPV RNA, red pen indicates pooled serum with detectable APPV RNA, and white pen indicates no detectable APPV RNA.

Atypical porcine pestivirus pen-level prevalence in the isolation nursery increased from 15 of 40 pens (37.5%) to 31 of 40 pens (77.5%) during the study period (Figure 4). Randomly selected pig prevalence increased from 4 of 20 pigs (20%) to 6 of 13 pigs (46%; Table 2). Of the five pen swab samples submitted, one, had detectable APPV RNA (Ct = 35.70) and another was suspect for APPV RNA (Ct = 37.64). During the finishing stage, eight of the 30 (27%) randomly selected pigs had detectable serum APPV RNA for the first sampling date but none were positive at the second sampling date (Table 3). During the finisher phase, on both sampling dates, all oral fluid samples had detectable APPV RNA.

**Table 2:** Detectable atypical porcine pestivirus (APPV) RNAemia status for 4 pens in an isolation nursery barn on two separate sampling dates.

	**Sampling day**	
**Item**	**8/20/2020**	**9/30/2020**
Pen 11		
Pig 173	Positive-24.38	-
Pig 188	ND	ND
Pig 47	ND	-
Pig 104	ND	Positive-26.99
Pig 37	ND	-
Pen 16		
Pig 70	ND	-
Pig 110	Positive-24.66	-
Pig 36	ND	ND
Pig 54	ND	ND
Pig 137	ND	Positive-26.32
Pen 28		
Pig 96	ND	Positive-30.46
Pig 123	ND	Positive-31.78
Pig 105	ND	-
Pig 168	Positive-30.08	ND
Pig 29	ND	Positive-35.33
Pen 38		
Pig 181	ND	Positive-33.14
Pig 184	ND	ND
Pig 182	ND	ND
Pig 69	Positive-33.01	-
Pig 12	ND	ND

If the individual pig's results had detectable APPV RNA, the cycle threshold value is given after the hyphen. ND = non-detectable APPV RNA. A dash indicates the individual pig was not present for the second sampling date. Pigs were part of a concurrent research trial and one pig pen from every pen were randomly selected to be euthanized.

**Table 3:** Detectable atypical porcine pestivirus (APPV) RNA for 30 individual pig serum on different sampling dates.

	**Sampling day**	
**Item**	**12/3/2020**	**1/16/2021**
**Serum**
1	Positive-36.02	ND
2	Positive-30.89	ND
3	ND	ND
4	ND	ND
5	ND	ND
6	ND	ND
7	ND	ND
8	ND	ND
9	ND	ND
10	Positive-35.67	ND
11	ND	ND
12	ND	ND
13	ND	ND
14	ND	ND
15	ND	ND
16	ND	ND
17	ND	ND
18	ND	ND
19	ND	ND
20	ND	ND
21	ND	ND
22	Positive-34.81	ND
23	ND	ND
24	ND	ND
25	Positive-36.29	ND
26	Positive-32.19	ND
27	Positive-37.24	ND
28	Positive-35.24	ND
29	ND	ND
30	ND	ND
**Oral fluid**
1	Positive-25.62	Positive-23.36
2	Positive-21.90	Positive-25.44
3	Positive-24.78	Positive-23.42
4	Positive-28.06	Positive-22.06
5	Positive-25.21	Positive-28.67
6	Positive-20.35	Positive-25.52
7	Positive-19.80	Positive-27.66
8	Positive-20.71	Positive-24.14
9	Positive-21.80	Positive-24.36
10	Positive-21.68	Positive-21.40

If the individual pig's results had detectable APPV RNA, the cycle threshold value is given after the hyphen. ND, non-detectable APPV RNA.

## Discussion

Atypical porcine pestivirus was identified, unintentionally, in the US in 2015 when samples were submitted for whole genomic sequencing for porcine respiratory and reproductive syndrome virus (1). The implications of this virus for swine production systems was uncertain until research established that inoculation of dams with APPV could results in clinical cases of CT and SL in piglets (2, 3, 6). However, even with considerable amounts of research-based evidence to suggest that CT and SL reside by weaning, there are inconsistent management strategies existing in commercial swine production systems for these piglets (17). For example, some production system elect humane euthanasia of all affected piglets while other production systems elect to let these piglets mature to weaning and re-evaluate proper management at that time. There is not a single best solution as to what works best for a production system but as understanding of this virus continues to grow, it's pivotal to ensure that swine production systems receive the most science-based support to avoid unnecessary piglet mortality (17). Additionally, CT/SL piglets that look seemingly normal can also be a source of virus to other naïve pigs resulting in continuous exposure of APPV within a production system (10). This is incredibly important to consider, especially in livestock intended for breeding purposes. However, there are gaps in understanding as to which samples to take, when to take them, and the availability of diagnostic tests. Some research work has looked at viral presence of APPV RNA in conjunction with APPV antibodies but this was a relatively small sample size and tests utilized are only available in research settings (18). Therefore, when a commercial farrow-to-finish production site observed an increased prevalence of APPV, there was an opportunity to understand the introduction of APPV onto the farm but also learn if diagnostic tests are available to evaluate long term APPV prevalence within a production flow of asymptomatic pigs.

One reason for increased appearance of CT/SL in litters from gilts is decreased prior exposure to pathogens (2, 3, 6). For this study, the similar clinical prevalence in sows and gilts indicated both groups had no previous exposure to APPV. However, after 2 months, gilt litters were primarily affected with clinical signs suggestive of APPV but by the end of the research period, clinical signs suggestive of APPV were not observed for any gilt or sow during lactation. These findings coupled with previous studies suggest an appropriate acclimation period for naïve females would decrease the likelihood of CT/SL appearance in their litters. Research has found that APPV RNA can be detected in boar semen and persist in boar reproductive tissues for long periods of time (2, 5, 19). The findings from this study in conjunction with other research indicate biosecurity measures such as screening of incoming semen would help to minimize the risk of APPV introduction and identify boars that are shedding APPV thereby serving as a potential source of APPV to other animals.

Of the randomly selected pigs for this study, no individual was positive for APPV at both sampling time-points potentially indicating transient infections. Pig identification numbers were obtained from finisher pigs to retrospectively analyze if they had been housed in pens that had detectable APPV RNA in the isolation nursery. Of the 30 randomly selected finisher pigs, only 15 of these pigs were bled in the isolation nursery, and these pigs were all housed in pens that had no detectable APPV RNA in the isolation nursery. These findings suggest the animals, within this population, housed in APPV negative pens were infected at a later age and maintained an asymptomatic infection for some time. These findings are similar to those in previous studies (2, 10). This study also tested oral fluids for detection of APPV and found they were an adequate means of APPV detection. Others have found the pig's saliva to be a source of APPV shedding implicating the potential for oral fluids to serve as a potential diagnostic sample (4, 10). In the current investigation, population-based oral fluid samples contained detectable RNA through the end of the finishing phase even after RNAemia was no longer detected in the subset of the population which was individually sampled. Thus, this data supports that oral fluids may be a useful diagnostic technique to detect APPV in a population of pigs. Further research should focus on understanding the duration of RNAemia in relation to shedding of virus through oral fluids to understand the potential utility of the oral fluid sampling diagnostic technique and the potential application of this technique for clinical decision making.

This study also found detectable APPV RNA in the environment after the isolation nursery was cleaned and disinfected. While this finding does not suggest this APPV may infect the subsequently-housed animals, it suggests the disinfection procedures used in this facility may not be sufficient to properly rid the virus from the environment. Others have reported that disinfection is an important part of control and prevention programs for APPV (4). Our findings suggest enhanced cleaning and disinfection procedures are required to eliminate all APPV RNA from the environment.

The data for this study are limited to one production site. Ideally more production sites would have been used to increase the sample size but the objective of this study was to identify introduction of APPV onto a naïve farm and understand the prevalence of APPV within the asymptomatic pigs. Furthermore, the diagnosis of APPV was made based on presence of RNAemia in a clinically affected piglet, case history and clinical presentation consistent with this diagnosis, and through ruling out other pathogens through metagenomic sequencing. Given this was a production facility which elected not to euthanize any clinically affected pigs for the purpose of histopathological analysis, the primary diagnosis was established based on molecular diagnostic techniques. Future research describing increased incidence of APPV cases in commercial swine facilities similar to that described in the current investigation should describe histopathological and serological findings as well as diagnostic capabilities improve over time. This work hopes to serve as a guide for other swine production systems when faced with a recent onset of CT or SL with unexplainable cause and elaborate on diagnostic tests available to aid in the investigative efforts.

In summary, APPV detected in a piglet with CT was similar to virus found in incoming semen, indicating that semen doses could serve as a route of APPV introduction onto the commercial farrow-to-finish farm. Further sample analysis and characterization would be needed to definitively conclude the origin of the APPV introduction within this farm. This study also found that APPV was detectable in populations of pigs without clinical signs of APPV indicating that these pigs could be a potential route of introduction for other naïve pigs. More evidence is needed to fully understand viral dynamics of transiently and persistently infected pigs and how this contributes to susceptible breeding livestock in swine production systems.

## Data availability statement

The datasets presented in this study can be found in online repositories. The names of the repository/repositories and accession number(s) can be found below: https://www.ncbi.nlm.nih.gov/genbank/, ON651441, ON651443, and ON651442.

## Ethics statement

The animal study was reviewed and approved by Kansas State University Institutional Animal Care and Use Committee.

## Author contributions

GH, CJ, JW, CP, TP, and JG contributed to conception and design of the study. GH organized the database. GH, RP, and JG performed the data analysis and interpretation. GH wrote the first draft of the manuscript. All authors contributed to manuscript revision, read, and approved the submitted version.

## Funding

This work was supported by the Kansas State University Swine Teaching and Research Center for diagnostic purposes.

## Conflict of interest

The authors declare that the research was conducted in the absence of any commercial or financial relationships that could be construed as a potential conflict of interest.

## Publisher's note

All claims expressed in this article are solely those of the authors and do not necessarily represent those of their affiliated organizations, or those of the publisher, the editors and the reviewers. Any product that may be evaluated in this article, or claim that may be made by its manufacturer, is not guaranteed or endorsed by the publisher.
